# Learned Value Magnifies Salience-Based Attentional Capture

**DOI:** 10.1371/journal.pone.0027926

**Published:** 2011-11-21

**Authors:** Brian A. Anderson, Patryk A. Laurent, Steven Yantis

**Affiliations:** Department of Psychological and Brain Sciences, Johns Hopkins University, Baltimore, Maryland, United States of America; Kyushu University, Japan

## Abstract

Visual attention is captured by physically salient stimuli (termed *salience-based attentional capture*), and by otherwise task-irrelevant stimuli that contain goal-related features (termed *contingent attentional capture*). Recently, we reported that physically nonsalient stimuli associated with value through reward learning also capture attention involuntarily (Anderson, Laurent, & Yantis, *PNAS*, 2011). Although it is known that physical salience and goal-relatedness both influence attentional priority, it is unknown whether or how attentional capture by a salient stimulus is modulated by its associated value. Here we show that a physically salient, task-irrelevant distractor previously associated with a large reward slows visual search more than an equally salient distractor previously associated with a smaller reward. This magnification of salience-based attentional capture by learned value extinguishes over several hundred trials. These findings reveal a broad influence of learned value on involuntary attentional capture.

## Introduction

Objects in the visual world compete for perceptual representation in the mind and brain. Selective attention resolves this competition, biasing perception in favor of behaviorally relevant and salient stimuli [1]–[4]. Because perception is limited in its representational capacity, which stimuli are selected by attention has important implications for the survival and well-being of an organism.

Attentional selection can proceed either voluntarily, according to context-specific goals and priorities, or involuntarily, according to the physical properties of a stimulus within a given task context. When a stimulus is selected via attention involuntarily, that stimulus is said to have captured attention. Attentional capture can be adaptive when a stimulus signals danger or opportunity [5], but comes at a cost in performance when those stimuli distract from ongoing goal-related processes.

It is well established that both physical salience and ongoing task goals influence the attentional priority of a stimulus. Physically salient but task-irrelevant stimuli slow visual search for a target in a spatially-specific manner (e.g., [6]–[10]); this is termed *salience-based attentional capture*. Irrelevant stimuli possessing goal-related features also capture attention involuntarily; for example, a red distractor captures attention when the searched-for target stimulus is partly defined by the color red [11], [12]. This is termed *contingent attentional capture* [13].

Salience-based and contingent attentional capture are known to jointly determine attentional priority. For example, contingent attentional capture is more pronounced for more salient distractors, even when the target of visual search is nonsalient [14]. These and related findings suggest that stimulus salience and ongoing task goals have a combined influence on attentional priority.

Physical salience and goal-relevance are not the only properties that influence attentional priority, however. A growing body of evidence has established that reward-related stimuli compete effectively for perceptual representation [15]–[27]. Attention to reward-related stimuli is often explicitly a behavioral goal; it is therefore necessary to design task contingencies such that it is possible to distinguish between the voluntary and involuntary deployment of attention to valuable stimuli [28].

Recently, we reported that otherwise nonsalient and task-irrelevant stimuli that had previously been associated with reward capture attention involuntarily [29]. In a training phase, participants received a monetary reward for identifying an oriented bar contained within a target stimulus that was unpredictably red or green; the target appeared in an array of other differently-colored items. Both high and low amounts of reward were given as feedback following each trial; one target color was associated with a high probability of a large reward while the other was associated with a high probability of a small reward. Thus, the amount of reward delivered on a given trial was not associated with a particular motor response, but was probabilistically related to the color of the target stimulus. Following the training phase, participants engaged in a test phase in which they searched for a shape-singleton target in extinction; no rewards were given, and color was irrelevant to the task. On half the trials, one of the nontarget items was rendered in the color of a formerly rewarded stimulus. Critically, the nontarget items were all differently colored, making the shape-singleton target the most physically salient stimulus in the display. The results revealed that distractors rendered in formerly rewarded colors consistently slowed visual search in a spatially-specific manner, even though they were nonsalient, task-irrelevant, and did not share any identifying features in common with the target [29]. This finding demonstrates that valuable stimuli capture attention involuntarily, in a manner that is distinct from other mechanisms of attentional control.

Although there is ample evidence that salience-based and contingency-based mechanisms of attentional control jointly determine attentional priority, it is unknown whether or how salience-based attentional priority is modulated by the learned value of a stimulus. This is an important question, as ecologically valuable stimuli are often visually salient. One possibility is that the attentional priority of a salient stimulus cannot be modulated by learned value, either because its priority is already maximal or because value and salience provide redundant information about attentional priority. Another possibility, however, is that stimulus salience and stimulus value combine to determine attentional priority, much as salience increases the attentional priority of goal-related stimuli in contingent capture [14]; this would result in a value-driven increase in attentional priority above and beyond that afforded by physical salience alone. Such a value-driven increase in attentional priority could either operate at the level of selection, effectively increasing salience, or at the post-selection level by prolonging attentional dwell time following purely salience-driven capture. In the present study, we distinguish between these two competing possibilities, and conclude that stimulus salience and stimulus value have combined effects on attentional priority.

## Experiment 1

**Figure 1:**
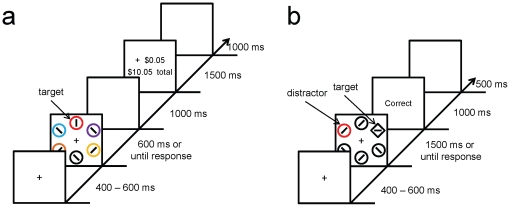
Behavioral Task. Sequence of events and time course for a trial during training (*a*) and at test (*b*) in Experiment 1.

The design of Experiment 1 was similar to that of Anderson et al. [29]. During the training phase, participants searched for a red or green target among differently colored nontargets (Figure 1a), and received visual feedback at the end of each trial indicating a monetary reward for a correct response; one of the two colors was associated with a high reward and the other with a low reward. During the test phase, which utilizes a variant of the additional singleton paradigm (e.g., [6], [9], [30]), participants searched for a unique shape in an array of usually all white elements (Figure 1b). On half the trials, one of the nontarget elements, the *distractor*, was rendered in red or green. Participants were informed that color was irrelevant and should be ignored, and the target was never red or green. No reward was provided during the test phase.

Color-singleton distractors capture attention robustly in a shape-search task by virtue of their physical salience (e.g., [6], [9], [30]). Our primary focus here was to determine whether a salient high-value distractor would capture attention more robustly than a salient low-value distractor. If salience completely dominates value, then high- and low-value color singletons should produce similar slowing in visual search. If learned value combines with physical salience, then the formerly high-reward distractors should slow responses more than formerly low-reward distractors.

### Materials and Methods

#### Participants

Eighteen participants were recruited from the Johns Hopkins University community. All were screened for normal or corrected-to-normal visual acuity and color vision. Participants were provided monetary compensation that varied between $21 and $28 (mean = $25.22), depending on their accuracy. All participants read and signed an informed consent form prior to participating in the experiments. Throughout the research project leading to this publication, the rights of the participants were protected and the applicable guidelines concerning the use of human subjects for the purposes of research were followed. The study was approved by the Johns Hopkins University Institutional Review Board.

#### Apparatus and Stimuli

A Mac Mini equipped with Matlab software and Psychophysics Toolbox extensions was used to present the stimuli on a Dell P991 monitor. The participants viewed the monitor from a distance of approximately 50 cm in a dimly lit room.

The sequence of events and time course for the training and test phases are shown in Figure 1a and 1b, respectively. Each trial consisted of three displays: a fixation display, a search display, and a feedback display. During both the training and test phases, the fixation display consisted of a white fixation cross (.5°×.5° visual angle) presented in the center of the screen against a black background, and the search display consisted of the fixation cross surrounded by six shapes (2.3°×2.3° visual angle) placed at equal intervals along an imaginary circle with a 5° radius.

Training Phase: During the training phase, the six shapes that comprised the search display were all circles of different colors (red, green, blue, cyan, pink, orange, yellow, and white). Targets were defined as either a red or green circle, one of which was presented on every trial in a randomly-selected location. Inside the target shape, a white line segment was oriented either vertically or horizontally, and inside each of the nontarget shapes, a white line segment was tilted at 45° to the left or to the right. The feedback display informed participants of the reward earned on the current trial, as well as total reward accumulated thus far.

Test Phase: During the test phase, the search display consisted of a white circle among white diamonds or a white diamond among white circles, and the target on each trial was defined as the unique shape. On a randomly-selected half of the trials, one of the nontarget elements, the *distractor*, was rendered in red or green (equally often). The feedback display at test only informed participants whether their response on the current trial was correct.

#### Design

The experiment consisted of a single session of 1008 training trials followed by 480 test trials. Participants were provided with 50 practice trials prior to the training phase, and 20 practice trials prior to the test phase. After every 100 trials and between the two phases, participants were provided with a short break. Target identity, target location, distractor color, and distractor location were fully crossed and counterbalanced, and trials were presented in a random order.

Correct responses in the training phase were followed by visual feedback indicating monetary reward. High-reward targets were followed by high-reward feedback ($0.05) on 80% of trials and low-reward feedback ($0.01) on the remaining 20%; for low-reward targets, the percentages were reversed. High-reward targets were red for half of the participants, and green for the other half. No reward feedback was provided during the initial practice block, and no reward feedback was provided during the test phase. Upon completion of the experiment, participants were given the cumulative reward they had earned.

#### Procedure

Each participant performed the experiment individually over the course of a single two-hour session. Each session took place inside a dimly lit laboratory room. The experimenter familiarized all participants with each task by providing written and oral descriptions of the stimuli and procedures. Participants were instructed to respond “as quickly as possible while minimizing errors.”

Each trial began with the presentation of the fixation display for a randomly varying interval of 400, 500, or 600 ms. The search display then appeared and remained on screen until a response was made or the trial timed out. The training task was performed under time pressure, with trials terminating after 600 ms; during test, time pressure was lifted by lengthening this time limit to 1500 ms.

Participants made a forced-choice target identification by pressing the “z” and the “m” keys for the vertically and horizontally orientated targets, respectively. Response time (RT) was measured from the onset of the target display until a response was made or the trial timed out. The computer emitted a 500 ms 1000 Hz feedback tone to inform the participant when a trial timed out. Only correct responses were included in the analysis, and all RTs more than three standard deviations above and below the mean of their respective conditions were excluded from the analysis.

### Results and Discussion

**Figure 2:**
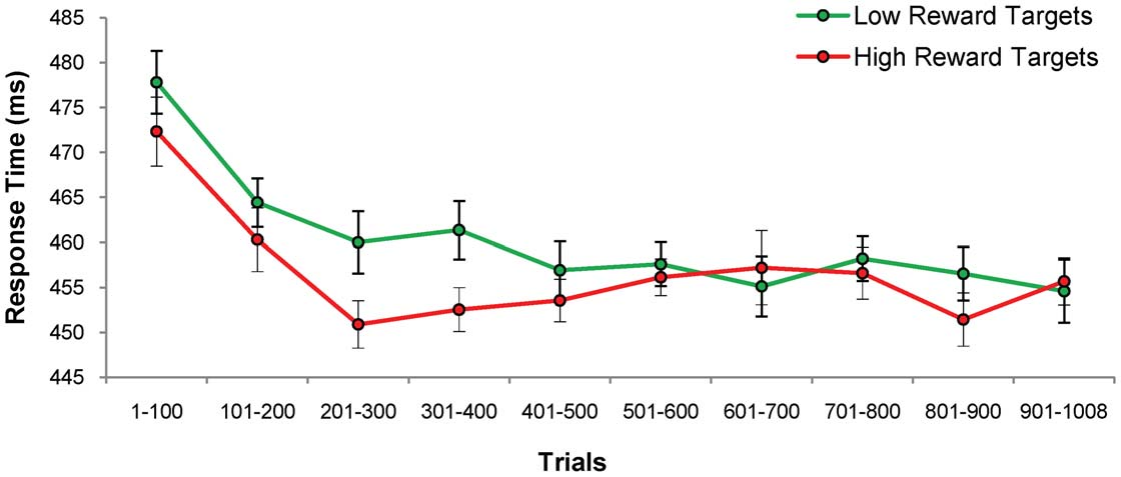
Behavioral Results for the Training Phase of Experiment 1. Mean response time ± within-subjects s.e.m. for high- and low-reward targets over the course of the training phase. Only the main effect of trial block was significant [*F*(9,153) = 4.92, *p*<.001, η*_p_* ^2^ = .224].

During training, mean RT to high- and low-reward targets did not differ significantly, although there was a trend toward faster responses to the target color associated with high reward, suggesting increased attentional priority [mean difference = 3.4 ms, *t*(17) = 1.57, *p* = .135]. To assess how the effect of reward on target selection changed over the course of the training phase, we analyzed the data from the training phase separately in ten bins of roughly 100 trials each. There was no interaction between reward and trial bin [*F*(9,153) = 1.43, *p* = .179], indicating that the influence of reward on RT did not change significantly over time. The main effect of trial bin was significant [*F*(9,153) = 4.92, *p*<.001, η*_p_* ^2^ = .224], however, showing that participants generally responded faster with more experience. The data for the training phase are presented in Figure 2.

**Figure 3:**
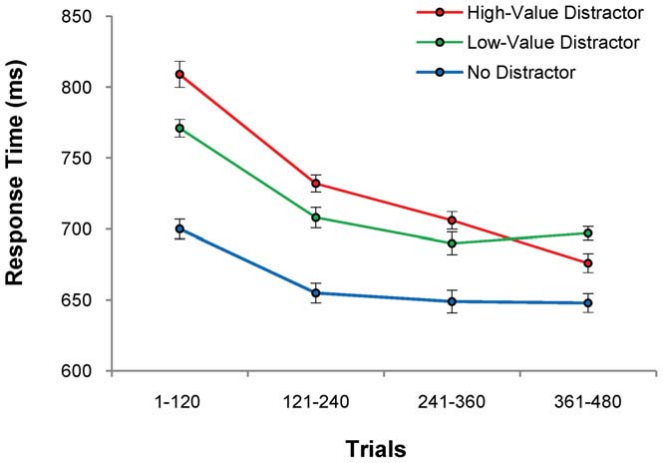
Behavioral Results for the Test Phase of Experiment 1. Mean response time ± within-subjects s.e.m. for each distractor condition over the course of the test phase. The difference in RT on trials containing a high-value vs. a low-value distractor represents the effect of learned value on salience-driven attentional capture.

Of particular interest were the data from the test phase. Reward-color mapping (i.e., red vs. green as the high-reward color in the training phase) did not interact with the effect of value on performance in the test phase (*F*<1), so further analyses collapsed across color. Response times (RTs) in the test phase differed significantly in the three distractor conditions [Figure 3, *F*(2,34) = 48.57, *p*<.001, η*_p_* ^2^ = .741]. Planned comparisons confirmed that both the high-value and low-value distractors slowed RT compared to when no distractor was presented [*t*(17) = 8.45, *p*<.001, *d* = 1.99 and *t*(17) = 6.31, *p*<.001, *d* = 1.47, respectively]. This replicates many previous demonstrations of attentional capture by irrelevant but physically salient feature singletons (e.g., [6], [7]).

We next examined the effect of reward history on performance in the test phase. High-value distractors slowed RT significantly more than did low-value distractors [*t*(17) = 3.37, *p* = .004, *d* = .81]. This modulation of attentional capture by reward history cannot be attributed to differences in physical salience, and occurred despite the irrelevance of the color items to the shape-search task. To assess how the effect of reward history on attentional capture changed over the course of the test phase, we analyzed the data from the test phase separately in four equally-sized 120-trial bins. The effect of learned value on performance gradually extinguished over the course of the unrewarded test trials, as revealed by a linear trend in the difference between RTs for high- and low-value distractor trials over trial bin [*F*(1,17) = 17.22, *p* = .001, η*_p_* ^2^ = .503]. There was no significant difference in error rates between the three distractor conditions (Table 1, F<1).

**Table 1:** Response times (in milliseconds) and error rates by distractor condition for Experiments 1 and 2.

Distractor Condition inExperiment 1			Distractor Condition inExperiment 2		
None	Low-Value	High-Value	None	Non-Target Colored	Target Colored
655(5.5)	710(3.9)	728(3.8)	588(3.6)	632(4.1)	634(4.8)
.09(.003)	.10(.004)	.10(.005)	.11(.003)	.13(.005)	.13(.005)

Error terms, in parentheses, reflect the within-subjects standard error of the mean (s.e.m.).

These results reveal that learned value magnifies attentional capture by salient stimuli. As the learned stimulus-value associations extinguished in the absence of reward, so did the effect of reward history on performance. However, extinction occurred gradually over many trials, resulting in a robust effect of prior reward on involuntary attention allocation for the first several hundred trials of the test phase. Taken together, these results provide strong evidence that learned value can magnify the effect of physical salience on attentional priority.

## Experiment 2

Despite the fact that attentional capture in Experiment 1 was significantly modulated by value, it could be that the effect of value on salience-based attentional capture was not critically dependent upon a learned association between stimuli and prior reward. Instead, it is possible that participants continued to maintain a search set for the training-phase target colors even in the test phase. Although it is known that participants can rapidly adjust task-related attentional priorities with changing task demands [31], former targets can continue to draw attention under certain conditions [32], [33]. Thus, it is important to rule out this possible explanation of our results.

We tested eighteen new participants who engaged in a training phase that was similar to that used in Experiment 1, with two critical differences. First, no reward feedback was provided during training or at any point during the experiment; instead, participants were compensated with a flat rate that matched the average earnings of participants in the main experiment ($25). Second, targets were now either red or blue (with green occurring as one of the nontargets) for half of the participants, and green or blue (with red occurring as one of the nontargets) for the other participants. The test phase for all participants was identical to that of Experiment 1. Thus, in the test phase, one color-singleton distractor had been a target color during the training phase, and the other color-singleton distractor had always been a nontarget color. If persisting priority for a former target color alone drove our main findings, we would expect an equally large – or indeed even larger – difference in RT on trials containing the color distractor that was used as a target during training versus trials containing the color distractor that was never used as a target during training.

### Materials and Methods

#### Participants

Eighteen participants were recruited from the Johns Hopkins University community. All were screened for normal or corrected-to-normal visual acuity and color vision. Participants were compensated with $25. None of the participants had participated in Experiment 1.

#### Apparatus and Stimuli

The apparatus and stimuli were identical to Experiment 1 with the following exceptions. Targets during training were either a blue or green circle (for half of the participants), or a blue or red circle. On half of the trials containing each target color, one of the nontarget-colored items was colored either red (for participants searching for green and blue targets) or green (for participants searching for red and blue targets). The feedback display during training only informed participants whether their previous response was correct.

#### Design and Procedure

The design and procedure were identical to Experiment 1, with the exception that no monetary reward feedback was provided.

### Results and Discussion

**Figure 4:**
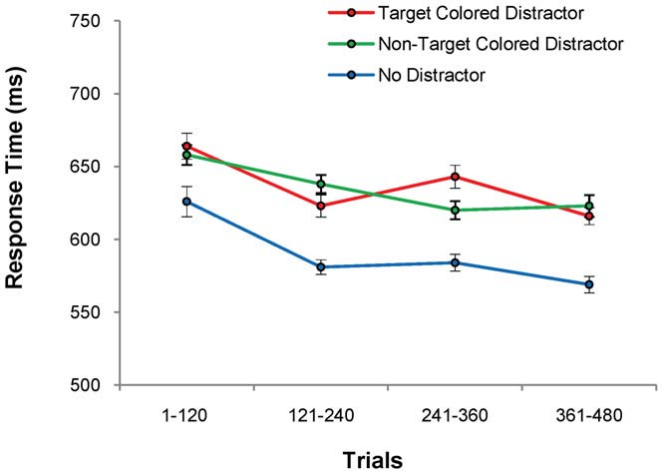
Behavioral Results for the Test Phase of Experiment 2. Mean response time ± within-subjects s.e.m. for each distractor condition over the course of the test phase.

Distractors at test were classified as being either the color of a former target or the color of a former nontarget. During the test phase, responses were significantly slowed by both former target-colored and former nontarget-colored distractors [Figure 4, *t*(17) = 7.27, *p*<.001, *d* = 1.71 and *t*(17) = 6.13, *p*<.001, *d* = 1.44, respectively]. However, we observed no difference in RT between those two distractor conditions [Table 1, *t*(17) = 0.34, *p* = .740]. The magnitude of slowing caused by the former target color distractors did not decrease over the course of the test phase (*F*<1), in contrast to Experiment 1. As in Experiment 1, there was no significant difference in error rates among the three conditions [Table 1, *F*(2,34) = 2.20, *p* = .127].

The slowing caused by the high-value distractor in Experiment 1 was significantly greater than that caused by the former target-colored distractor in Experiment 2 [mean difference = 27 ms, *t*(34) = 2.29, *p* = .025, *d* = .79]. This outcome demonstrates that value associations are necessary to produce the modulation of distraction observed in Experiment 1, and that this modulation cannot be explained merely in terms of a persisting intention to search for former targets.

## General Discussion

It is well established that physical salience and ongoing task goals influence attentional priority involuntarily (e.g., [6], [13]), and recent research indicates that the learned value of a stimulus also plays a direct role in determining its attentional priority [29]. Although salience-based and contingency-based mechanisms of attentional control are known to jointly influence attentional priority, it is unknown whether attentional priority to salient stimuli is similarly modulated by learned value. In the present study, we addressed this question and show that the physical salience and learned value of a stimulus have a combined effect on attentional priority, with learned value increasing attentional priority above and beyond the level afforded by salience alone.

Our results demonstrate that a salient but otherwise neutral stimulus, when previously associated with high reward, magnifies distraction even after that stimulus no longer predicts reward. This finding cannot be attributed to differences in physical salience, and Experiment 2 rules out persisting intention to search for a former target as an explanation. Instead, our results reveal a broad influence of learned value in determining attentional priority, one that combines with salience-based mechanisms of attentional control such that more valuable stimuli receive increased attentional priority in addition to the priority afforded by their physical salience.

Navalpakkam et al. [22] showed that attentional selection reflects an optimal weighting of the conspicuity of a stimulus afforded by its physical salience and its associated reward value, suggesting that value-based and salience-based attentional priority can be independently adjusted according to the relative importance of each factor given the demands of the task. There are multiple mechanisms through which value and salience might be combined in order to jointly determine attentional priority in this way. One is that learned value directly modulates the visual salience or pertinence [34] of reward-associated stimulus features, thereby increasing their attentional priority. This possibility is supported by evidence showing that reward-associated stimulus features are represented more robustly in early visual areas of the brain [25], [26]. Another possibility is that the learned value of stimuli increases attentional dwell time – that is, the time required to disengage attention after it has been captured [7], [35], [36]. However, we have previously shown that the learned value of stimuli is sufficient to drive attention allocation in the absence of prior attentional capture on that trial by salience or goal-relevance; similar value-driven slowing of response time has been reported for nonsalient stimuli as well [29]. Thus, although a post-capture dwell time account of value-based attentional priority is plausible and cannot be ruled out in the present study, it does not provide a complete account of how learned value is known to influence attention. A third possibility is that associating targets with value causes individuals to perseverate with goal-related priorities that have been rewarded previously. This could be likened to contingent attentional capture on the basis of a reward-motivated goal state that cannot be easily overcome by virtue of its association with reward. However, because attention to valuable stimuli ran counter the goals of the task in the test phase, it is clear that the reported effects on attentional priority reflect an involuntary modulation of attentional priority by previous reward history.

Our results contribute to a growing body of work that highlights an important role for reward in perception and attention [15]–[27], [29]. Attentional priority to reward-related stimuli will often be adaptive, serving to maximize reward procurement. However, an inability to ignore formerly rewarding stimuli that run counter to current behavioral goals, such as desired abstinence from a drug of abuse, can be highly maladaptive. In this way, the value-based modulation of attention may play a key role in a variety of clinical syndromes in which both attention and reward have been critically implicated, including drug addiction [37]–[39], obesity [40], attention-deficit hyperactivity disorder [41], and obsessive-compulsive disorder [42]. All four of these conditions are highly comorbid [41], [42], suggesting a common underlying mechanism that may be related to susceptibility to the value-based modulation of attentional priority.
